# An evaluation and refinement of the “Hep B Story” app, tailored to meet the community’s cultural needs

**DOI:** 10.1186/s12913-024-11149-y

**Published:** 2024-06-07

**Authors:** Paula Binks, Sudharsan Venkatesan, Anngie Everitt, George Garambaka Gurruwiwi, Roslyn Gundjirryirr Dhurrkay, Sarah Mariyalawuy Bukulatjpi, Cheryl Ross, Tiana Alley, Kelly Hosking, Emily Vintour-Cesar, Melita McKinnon, Richard P. Sullivan, Joshua S. Davis, Marita Hefler, Jane Davies, On behalf of the Hep B PAST partnership

**Affiliations:** 1 Global and Tropical Health Division, Menzies School of Health Research, Charles Darwin University, Darwin, NT Australia; 2 Department of Infectious Diseases, Royal Darwin and Palmerston Hospitals, Northern Territory Health, Darwin, NT Australia; 3 Miwatj Health Aboriginal Corporation, Nhulunbuy, East Arnhem Land, NT Australia; 4 Public Health Directorate, Office of the Chief Health Officer, Northern Territory Health, Darwin, NT Australia; 5 Department of Infectious Diseases and Immunology, School of Clinical Medicine, St George and Sutherland Hospital, UNSW Medicine and Health, Sydney, NSW Australia; 6 School of Medicine and Public Health, University of Newcastle, Callaghan, NSW Australia

**Keywords:** Hepatitis B, Aboriginal and Torres Strait Islander peoples, Health literacy, Health education, Cultural safety, Electronic health apps, Shame and stigma, Women’s business

## Abstract

**Background:**

Hepatitis B is endemic amongst the Australian Aboriginal population in the Northern Territory. A participatory action research project identified the lack of culturally appropriate education tools and led to the development of the “Hep B Story” app in the Aboriginal language Yolŋu Matha. This paper describes a formal evaluation of the app’s first version, which informed improvements and translation into a further ten Aboriginal languages.

**Methods:**

The evaluation employed Participatory Action Research (PAR) principles to work within Indigenous research methodologies and prioritise Indigenous knowledge to improve the app iteratively. Semi-structured interviews and focus groups were conducted across the Northern Territory with 11 different language groups. Local Community Based Researchers and Aboriginal Research team members coordinated sessions. The recorded, translated conversations were transcribed verbatim and thematically analysed using an inductive and deductive approach.

**Results:**

Between November 2018 and September 2020, 94 individuals from 11 language groups participated in 25 semi-structured interviews and 10 focus groups. All participants identified as Aboriginal. Most participants felt the app would be culturally appropriate for Aboriginal communities in the Northern Territory and improve knowledge surrounding hepatitis B. The information gathered from these interviews allowed for identifying five main themes: support for app, relationships, concept versus language, shame, and perceptions of images, along with errors that required modification.

**Conclusions:**

A “real-life” evaluation of the app was comprehensively completed using a PAR approach blended with Indigenous research methods. This evaluation allowed us to develop an updated and enhanced version of the app before creating the additional ten language versions. An iterative approach alongside strong community engagement was pivotal in ensuring the app’s cultural safety and appropriateness. We recommend avoiding the use of knowledge-based evaluations in an Aboriginal setting to ensure relevant and culturally appropriate feedback is obtained.

**Supplementary Information:**

The online version contains supplementary material available at 10.1186/s12913-024-11149-y.

## Background

Chronic Hepatitis B (CHB) poses a significant global health challenge, impacting more than 290 million individuals [1], leading to serious complications like cirrhosis and liver cancer if left untreated [2]. In Australia, as of 2021, approximately 200,385 people were living with CHB [3]. Within the Northern Territory (NT), the prevalence of Hepatitis B Virus (HBV) is 1.73% [3], the highest in Australia. It has a disproportionate impact on Aboriginal and Torres Strait Islander populations, with prevalence of 6% [4–7]. While socioeconomic factors, healthcare access, and language barriers can present challenges [8], recent research shows that community-led, systematic approaches to CHB management improve the cascade of care in Aboriginal communities [9–12]. Early diagnosis through effective clinical management is crucial in mitigating the risk of liver cancer and associated mortality in people living with CHB [13]. With its diverse linguistic landscape boasting over 100 languages [14], the NT necessitates healthcare services tailored to Aboriginal and Torres Strait Islander communities’ unique needs. Addressing low CHB literacy and communication gaps between healthcare providers and Aboriginal and Torres Strait Islander people is essential for comprehensive CHB treatment [15–17]. In 2012, a senior Aboriginal Health Practitioner (author SB) identified a significant lack of CHB knowledge within remote Aboriginal communities, especially in conveying CHB information to clients who did not primarily speak English. This observation led to a collaborative Participatory Action Research (PAR) project involving Menzies School of Health Research (Menzies), the local community, and healthcare staff [16, 17]. The aim was to enhance health literacy among Aboriginal Australians in the NT and primary healthcare providers.

Due to the project’s location in the NT, with the predominant population primarily identifying as Aboriginal, the term “Aboriginal” will henceforth be used respectfully, recognising no Torres Strait Islander language groups were involved in the project.

The project was undertaken in three phases. Phase one (2012–2013) involved a qualitative inquiry exploring the knowledge, perceptions, and experiences of Aboriginal individuals and their healthcare providers in a remote community regarding hepatitis B. This study uncovered key themes, including limited biomedical knowledge, unfavourable perceptions of hepatitis B, and challenges related to communication and culture [16]. This research highlighted the importance of using an educational tool in a patient’s first language to develop successful treatment partnerships [16]. Community members expressed a preference for using electronic visual aids with simple language to enhance communication and promote health literacy specific to CHB [16].

Phase two (2012–2014) of the project involved the collaborative development of the “Hep B Story” app in consultation with the community members, as previously reported [17]. The app underwent multiple evaluations with community members, patients, and healthcare workers [17]. Aligning with World Health Organization (WHO) guidelines [18], the app’s content was translated into Yolŋu Matha, an Aboriginal language used in East Arnhem Land in the NT. The “Hep B Story” app was made available for free on the Apple App Store, Google Play store and the Menzies website in Yolŋu Matha and English. It is the first educational tool on hepatitis B created in an Aboriginal language.

The original plan to evaluate the “Hep B Story” app was through a pre-and-post-app knowledge questionnaire on two separate occasions: at a Viral Hepatitis Conference in Alice Springs, NT, and during the app’s launch in the community in East Arnhem Land, where it was initially developed. These preliminary “Hep B Story” app evaluations garnered overwhelmingly positive feedback. However, a notable discrepancy emerged between pre- and post-app knowledge questionnaire completion rates during the remote community launch day and the conference delegate group. The completion rates were significantly lower on the community launch day. The Menzies Infectious Diseases Indigenous Reference Group (IRG) was consulted to understand the underlying reasons in response to this difference. Feedback from the IRG indicated that “knowledge-based assessment” felt intimidating, even when presented in the local language. Consequently, the IRG recommended exploring alternative culturally appropriate methods to evaluate the app’s content in remote Aboriginal communities.

Phase three (2018–2023) constituted a component of a larger project known as Hep B PAST – A Partnership Approach to Sustainably eliminating Chronic Hepatitis B in the Northern Territory. The primary aim was to improve health literacy around hepatitis B within Aboriginal communities, individuals living with CHB and primary healthcare providers. This was facilitated by enabling people living with CHB and their communities to have access to culturally appropriate and effective education tools. To achieve this, the “Hep B Story” app was evaluated and refined before translation into an additional ten Aboriginal languages (Table 1.). This ensured that over 70% of the NT’s Aboriginal population would have access to hepatitis B education in their preferred language. The selection of these languages was guided by factors such as the number of speakers [14, 19–22] and the regions within the NT with a greater need for information due to the prevalence of CHB [3].

**Table 1:** Aboriginal Languages selected for translation [14, 19–22]

Language	No. of Speakers	Region of Northern Territory
Kriol	20,000	Katherine
Yolŋu Matha	6806	East Arnhem
Arrernte	5475	Alice Springs
Murrinh-Patha	3100	Wadeye
Pitjantjatjara	3000	Western Desert
Warlpiri	2509	Central
Tiwi	2102	Tiwi Islands
Kunwinjku	2000	West Arnhem
Anindilyakwa	1600	Groote Eylandt
Burarra	1000	Maningrida
Gurindji	900	Katherine West

With 11 Aboriginal languages and English, the app is a practical tool for Aboriginal Health Practitioners, Aboriginal Health Workers, Community Based Researchers, Doctors, Nurses, and other health care providers. Healthcare professionals can introduce patients to the “Hep B Story”, guiding them through the information and encouraging them to download it onto their personal devices for future reference. Community Based Researchers can also utilise the app for CHB-related research, contributing to community-wide education. It is hoped this will improve health literacy amongst CHB patients, community members and health care providers, especially in remote health clinics with high CHB prevalence.

## Methods

Before translating the “Hep B Story” into ten more languages, evaluating and refining the content was essential to ensure it was culturally appropriate for each language group. It is unsafe to assume that what is suitable for one language group will apply universally. Across Australia, Aboriginal cultures vary significantly, with different ancestral backgrounds shaping aspects of daily life such as kinship, language, art, law, and ceremonies [23]. Our initial literature search for a health app evaluation framework did not yield a structure suitable for our needs. Hensher et al. echoed this sentiment and identified a need for a health app evaluation framework to integrate the full range of evaluative criteria [24]. Guided by the Menzies Infectious Diseases IRG, we implemented a decolonising approach, employing a culturally appropriate methodology to evaluate the app extensively. We evaluated the app using an approach that considered Aboriginal Peoples’ holistic view of health, which includes the physical, social, emotional, cultural and spiritual domains of individuals, families and communities [25]. The PAR method facilitated the evaluation in a real-world setting to ensure that any feedback received was genuine and culturally relevant. We implemented Indigenous research methodologies, prioritising the Indigenous ways of knowing, doing, and being [26, 27]. We also involved Aboriginal researchers, stakeholders and community members in all aspects and stages of research planning and development, data collection, data analysis and dissemination of results as outlined in the CONSIDER statement (consolidated criteria for stengthening reporting of health research involving Indigenous peoples) [28]. This approach was regarded as the “right way” to evaluate the app by the participants involved in the study.

### Setting

This study was conducted in the Northern Territory (NT) of Australia, which has a population of 251,700 [29] and covers a region of approximately 1.34 million square kms [29, 30]. The NT encompasses the tropical Top End to the arid Central region. Numerous remote communities are accessible by road only during the dry season, becoming isolated during the wet season, where air travel is the primary means of access. Aboriginal languages are vital for communication within Aboriginal communities, playing a central role in cultural practices such as ceremonies and hunting. While many Aboriginal individuals are proficient in multiple Aboriginal languages, English proficiency varies. Literacy, particularly in English, is often limited in this predominantly oral culture [22].

### Researcher reflexivity

The team includes Aboriginal members (GG, RD, SB, CR, TA) representing the Yolŋu, Arrernte, Kaytetye, Alawa and Marra language and cultural groups. They bring expertise as clan leaders, Aboriginal Health Practitioners, Aboriginal Community Workers, and Researchers with strong NT family and community connections and provide significant cultural guidance for non-Aboriginal team members. The non-Aboriginal team members (PB, SV, AE, KH, EVC, MM, RS, JSD, MH, JD) offer substantial experience in Aboriginal clinical service delivery and/or research. The entire team follows Participatory Action Research (PAR) principles [31], fostering respect and inclusivity, collaborating across the whole research spectrum from setting research priorities, conceptualisation of projects, data collection and analysis and presentation and publication of results. The team members are dedicated to self-reflection and bias reduction efforts, as demonstrated in their work [9–12, 16, 17, 32–35].

### Ethical approval

Ethical approval was obtained from the Human Research Ethics Committee of the Northern Territory Department of Health and Menzies School of Health Research (NTHREC 2018–3240), (NTHREC 2018–3242).

### Participants

Purposive and network sampling methods were employed to identify individuals representing three specific target groups: Aboriginal Health Workers, CHB patients and community members. These groups were predetermined based on their potential to provide the most relevant feedback aligned with our objectives. The Aboriginal researchers purposefully selected community members to include a range of ages, genders, and family groups. Informed written consent was obtained from each participant in accordance with the Human Research Ethics Committee of the Northern Territory Department of Health and Menzies School of Health Research.

### Data collection and analysis

The “Hep B Story” was evaluated in two distinct stages. Stage one occurred in East Arnhem Land in the community where the app was initially developed and focused on the English and Yolŋu Matha versions. Stage two of the evaluation reviewed the English version of the app with each of the additional ten language groups; this involved travelling extensively across the NT. A mix of focus groups and semi-structured interviews (either individual or in a group according to participant preferences) were conducted throughout both stages.

Two experienced Aboriginal Community Based Researchers (RD, GG) coordinated the stage one evaluation together with senior Aboriginal Health Practitioner (SB) and non-Aboriginal team member (PB). Stage two was coordinated by Aboriginal researchers (CR, TA) and non-Aboriginal team member (PB).

The participants and Aboriginal members of the research team guided the method employed to collect data at each interview or focus group. Interviews and focus groups were conducted in a culturally safe manner (for example, being mindful of gender, skin groups and kinship relationships). An interview and focus group discussion guide was developed specifically for the evaluation (refer to supplementary file S2) and prompted team members to create conversations that explored the participants’ hepatitis B knowledge, thoughts about the content and cultural safety of the “Hep B Story” app, and attitudes toward biomedical health knowledge in a predominantly Western medical system. The process involved in-depth discussions of each app page, leading to numerous questions and extensive conversations. The interviews and focus groups took anywhere from 30 min to four hours, depending on the discussion generated by participants (e.g. storytelling or seeking a deeper understanding of hepatitis B from a medical perspective). These data collection activities occurred at community health clinics, community gathering places, and participants’ homes. Voice recorders were used to capture conversations, while some participants who preferred not to have their voices recorded provided answers on paper case report forms, which were subsequently summarised and verified with them. Every participant had the chance to use their preferred language, which ensured participants could express their ideas and concerns and ask questions with the assistance of interpreters. The recorded and translated conversations were then transcribed verbatim. Interview transcripts were analysed by Aboriginal and non-Aboriginal team members (SV, AE, SB, PB, CR, JD). Using grounded theory [36], the team members immersed themselves in the data through reading and listening to the recorded conversations and written responses. Microsoft® Excel® for Microsoft 365 MSO (version 2402) was utilised to organise and code the data. Each team member independently applied inductive and deductive coding [37] to the same transcripts before convening to deliberate on emerging themes. Deductive coding of the data enabled the exploration of hepatitis B knowledge, cultural safety, app content and attitudes towards biomedical health knowledge. Inductive coding aided in identifying areas requiring refinement within the app and other concerns surrounding the hepatitis B virus phenomenon. Despite minor variations, overall coding similarities were observed. Any discrepancies were discussed until consensus was achieved. Subsequently, the data were organised into distinct categories and themes. The COREQ (consolidated criteria for reporting qualitative research) checklist was utilised to guide reporting of results [38].

## Results

**Fig. 1:**
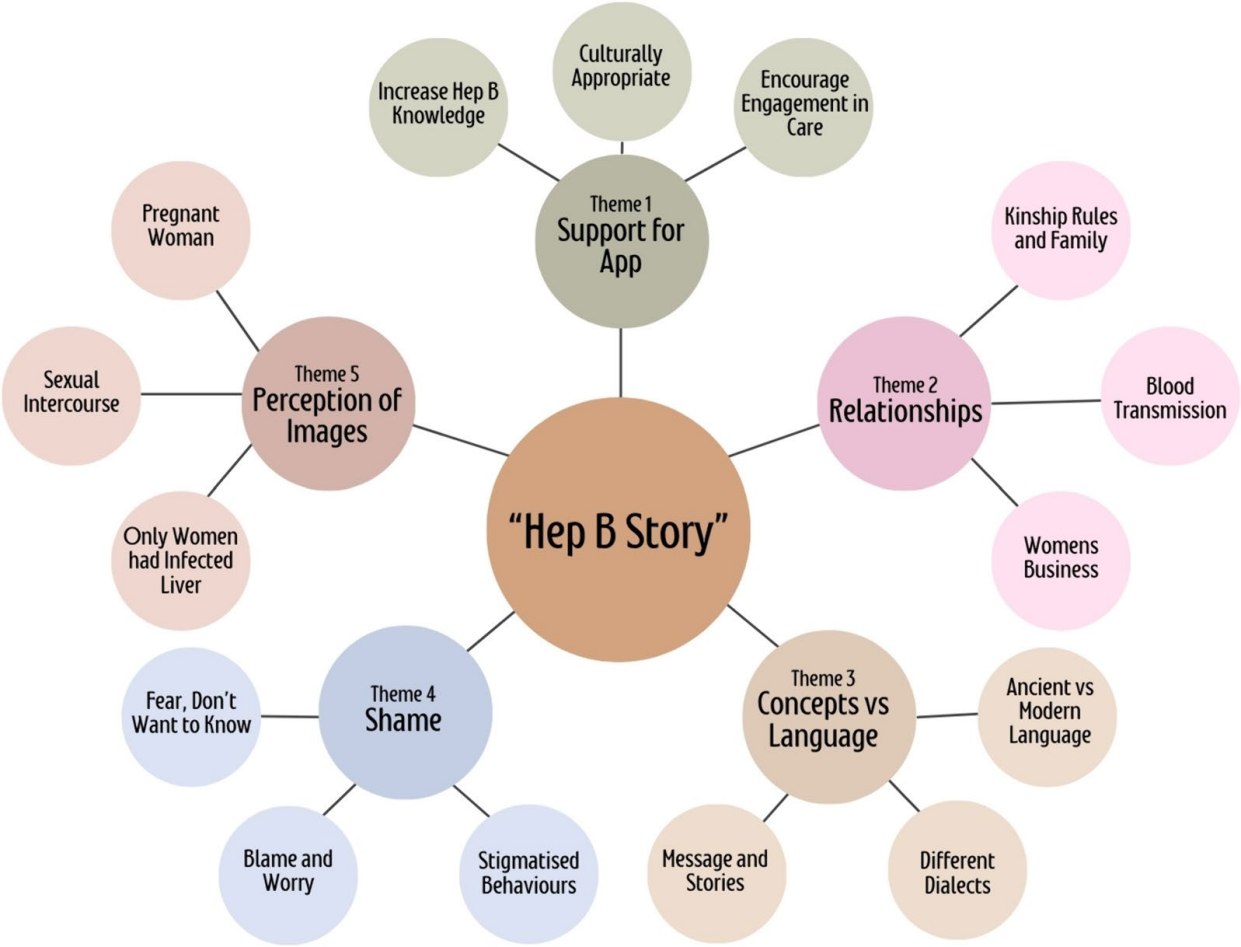
Thematic diagram representing the five distinct themes and their subthemes

Between November 2018 and September 2020, 94 individuals from 11 language groups participated in 25 semi-structured interviews and 10 focus groups. All participants identified as Aboriginal Australians. Five interrelated but distinct core themes were identified from the analysis of the data (Fig. 1): support for app, relationships, concept versus language, shame, and perceptions of images. Furthermore, we assessed the usability and functionality of the “Hep B Story” from observations made of the participants using the app in interviews and focus groups. Overall, feedback from all language groups was positive.

### Support for app

Very few of the participants had some prior knowledge of hepatitis B. Of those who had heard of hepatitis B, they felt their knowledge was inadequate.

“I haven’t heard of it, I heard different stuff but not hepatitis B”.

Aboriginal Community Member

“Well, I honestly didn’t know hep had anything to do with the liver. Honest to God, I did not know that”.

Aboriginal Community Member

One senior Aboriginal Health Practitioner acknowledged they had heard about hepatitis B while working at the local health clinic but didn’t have an in-depth understanding of the condition despite being required to assist with interpreting during clinical consultations.

“Yeah, that’s when the doctor came and saw me and wanted me to help to explain to this patient there about hep B, while I was interpreting, I was learning”.

Senior Aboriginal Health Practitioner

All participants felt that the “Hep B Story” app was helpful with respect to increasing knowledge about hepatitis B.

“Yeah, yo (Yolŋu Matha: yes), it help (sic) in Yolŋu Matha so they can understand quite well what the meaning of this hepatitis B and what is sickness and what you know”.

Aboriginal Community Member

When asked, most participants felt the app would be culturally appropriate for Aboriginal communities in the NT.

Yes, I reckon. To me, it’s appropriate. If it’s got all the pictures and showing them, you know, as you’re talking, it’s got all those pictures of it.

Aboriginal Health Practitioner

Most participants required no assistance using the app, as smartphone technology is commonplace in remote communities.

Yeah, it does make sense and it’s pretty easy in the long run. You can download it on your phone, and you know, we could explain that to countrymen and show them, if they’ve got an iPhone or whatever the phone, and then we can just download it.

Aboriginal Health Practitioner.

Several participants felt that the content in the app was excessive or took too long to go through and suggested creating an additional complementary smaller version of the app with key messages. Other suggestions were to make a video that could be played on the television in clinics or a small book.

“Don’t want it too long because people get bored, and they wander off”.

Aboriginal Community Member

In contrast, other participants felt there needed to be more information included in the app.

“Not enough, that’s what I think, yeah we need more information, we need to learn much more on that one”.

Aboriginal Community Member

The majority felt it would encourage people to present at the local health clinic for a “check-up”.

“See going through that (the hep B story), listening to it and the people I know and where they are actually make me think straight away to the clinic. It made me think of that straight away. It would make you want to go and see him (the doctor at the clinic) and ask him for a blood test, so it does make you want to go and do that”.

Aboriginal Community Member

Overwhelmingly, people felt that the information about hepatitis B was essential and intended to pass on their new knowledge to family and other community members.

“When I go home, I will tell my children about it. I want to explain to them.”

Aboriginal Community Member.

“Yeah, so then we can keep on going on and telling people. Yo, telling the story again and again”.

Aboriginal Community member.

All participants felt shame associated with CHB, but the app would help decrease the feelings of shame.

“Because it’s not a shame job, and you just need to take it (the app) out and go and promote it – you know, show pictures because that’s how our mob are – like to look at the pictures and know the story from that”.

Aboriginal Health Practitioner

Many participants felt some of the information in the app was inappropriate or threatening. This was explored further in the data generated from the interviews and focus groups.

### Relationships

The importance of relationships within Aboriginal communities was a prominent theme. It is essential to respect cultural norms around kinship, women’s business and interactions between males and females. Understanding, respecting, and behaving appropriately in accordance with these protocols is necessary to ensure cultural safety for Aboriginal people. Of note, kinship rules, discussion of blood-to-blood transmission between males and females and women’s business were raised consistently by participants as topics that could potentially offend when discussing the “Hep B Story”, highlighting the need to exercise care and consideration for kinship dynamics when broaching the subject of hepatitis B in a mixed-gender group.

Many participants felt discussions involving blood could only proceed with “right way” relationships. They should be told “man to man, woman to woman” and never told to a brother and sister simultaneously. Therefore, listening to the “Hep B Story” could cause offence if siblings heard the story together.

“Sister, brother, no good. Yo. Mother-in-law, son no good. Sister-in-law, brother no good.”

Aboriginal Community Member

“If you’re talking about blood, then it’s - you can’t talk to someone… Like you can’t talk to someone like your sister”.

Aboriginal Health Practitioner

**Fig. 2:**
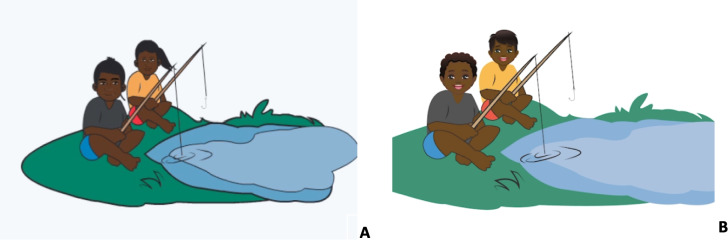
“The Fishing Story” tells the story of hep B transmission between a girl and boy, **A** original story (boy and girl), and **B** new story (two boys)

Another concern consistently raised by participants was the discussion of blood transmission between male and female persons. “The Fishing Story” tells the story of hepatitis B transmission between a little girl and boy. The little girl pricks her finger on a fishing hook, and a drop of blood lands on an open wound on the little boy’s leg, giving him hepatitis B. This story, while explaining how easy it is to get hepatitis B, was not appropriate as it discussed blood transmission between a male and female. The “Fishing Story” has since been modified to a story about two little boys (Fig. 2).

“I think because of the blood-to-blood thing, that does scare people, yeah, and can make you uncomfortable to talk about it”.

Aboriginal Health Practitioner

A recurring theme emphasised the notion that men should refrain from viewing or discussing “women’s business”, which pertains to such topics as pregnancy and childbirth. Despite the app having a dedicated section for women’s business, the sentiment remained that discussions surrounding these matters should solely involve females. Such topics are considered inappropriate for men to talk about, underscoring the cultural belief that they should be exclusively discussed among women.

“It’s just ladies, you know, have to talk about that one the most, not the mans”.

Aboriginal Health Worker

“The woman’s story. Yeah, some of like lady stuff. That, I mean, gender thing, like lady stuff”.

Aboriginal Health Practitioner

Despite the consensus that it was inappropriate for men to engage in the subject of women’s business, there was agreement that the information relating to hepatitis B transmission was important. This was especially relevant in the NT context, where the primary mode of hepatitis B transmission is in early childhood, including mother-to-child [32]. The app’s original design intentionally featured a dedicated section on women’s business, requiring deliberate selection to access the information, ensuring men would not accidentally encounter it. However, the “other ways to get hepatitis B” section of the app addresses mother-to-child transmission. After careful deliberation, participants concluded that despite potential discomfort for men, it was vital for them to understand all modes of hepatitis B transmission. Therefore, the mother-to-child transmission information should remain in the “other ways to get hepatitis B” section.

### Concept versus language

Several issues identified in the “Yolŋu Matha” version of the app, directly related to the complexities of translating concepts. These challenges stemmed from various factors. For instance, the use of ancient versus modern language, the differences between dialects and the utilisation of storytelling to convey words lacking direct equivalents in Aboriginal languages. Translating Western biomedical words and concepts into Aboriginal languages is extremely difficult. It requires intricate thought processes, bridging knowledge gaps and creating analogies for words without direct equivalents. A straightforward word-for-word translation of a script risks misinformation and loss of context and meaning. Ensuring accurate conceptual transmission often entailed crafting and sharing stories to convey the message correctly [11].

When a suitable modern language word or explanation was unavailable, a substitution from the ancient language was used. Consequently, the younger generation may need help understanding the word (and concept), requiring further explanation from the older generation.

“There’s two type of talking, yo. So, next generation has to know the older way talking. Because sometimes the younger one can’t understand the older word”.

Aboriginal community member

“It would help this one shame. The stigma about hep B. If we used that nowie (sic) dialect – the old language, people might understand – understand it you know. There’s no words in the new language to describe”.

Aboriginal Health Practitioner

Avoiding the transmission of incorrect messages proved more challenging than initially expected. Even within a single language group, multiple dialects exist, each with distinct differences.

That’s the problem with (location) itself – you know, the community of (location) is they’ve got – you know, you’ve fifty different dialects here in (location) itself. So, who do you approach to set this out to? You know.

Aboriginal Community Member

“East and central is more close together, so, I think – see, with western, it’s mostly just (community name) mob. The dialects, it’s similar, but they say it’s completely different. Yet east and central’s more the same. See, there’s central, and then there’s eastern central, and then there’s eastern, and there there’s southern, and that’s just one language”.

Aboriginal Community Member

Selecting the appropriate dialect to accommodate most individuals within a single language required considerable deliberation. This task was made more challenging by the realisation that even simple word choices could convey incorrect messaging and lead to the sharing of inaccurate stories.

Like I said, the dialects are similar, but there are some wording that means something else, so it might get lost in translation and mean something completely different.

Aboriginal Community Member

Extensive consultation was needed to ensure that the translated versions were medically and factually correct; this process took considerable time and effort.

Several inaccuracies were identified in the Yolŋu Matha version of the app. One error in the Yolŋu Matha version of the app was in the sentence, “virus is a medical word meaning tiny, invisible germ that needs to live inside a person, or animal, to stay alive”. The word “animal” was translated to “dog”, which offended several Yolŋu participants. This translation inadvertently implied that only humans or dogs could become infected with hepatitis B, thereby bringing shame. To address this issue, the word “animal” was removed from the English script before being translated into the other Aboriginal languages to prevent the problem from arising in any of the other ten languages.

“ Yeah. Some of the wordings - we don’t like… Yo. The dog one”.

Aboriginal community member

Although the language used in the app was designed to be non-threatening, once translated, there may have been unintended modifications to the contextual meaning of the translated version. The line “you can’t survive long without a working liver” became threatening when translated.

“Telling now (sic) person that he’s going to die in the app - I heard the stories there, you know. It’s just like a threat, eh?”.

Aboriginal Community Member

The issue of threatening language leading to fear was voiced by one focus group participant, who recounted the story of her close family members not engaging in care for their CHB due to fear.

“They (close family members) knew they had it but was too frightened to go to the hospital, some of them passed on, they passed away”.

Aboriginal Community Member

All errors in the Yolŋu Matha version were rectified. While these instances highlight the participant’s occasional offence at certain concepts and language used in some sections of the translated version of the “Hep B Story”, participants believed their family members needed to grasp this information, with all participants intending to share it.

### Shame

A lack of understanding about how hepatitis B is transmitted led to misconceptions and shame. Among the few individuals who possessed prior knowledge of hepatitis B, there existed deep-seated shame. For Aboriginal people, shame can disrupt cultural identity, leading to feelings of alienation from family and community [39]. Among the very few individuals who possessed prior knowledge of hepatitis B, transmission of the virus was associated with activities like intravenous drug use and having multiple sexual partners, which unfairly contributed to stigma.

“Most of my, you know, my mob, our mob doesn’t really know, only in the clinic, sort of thing. Yeah, they don’t really know the whole story about how it’s been passed. All they knew that, I think, just thinking back, all they knew was that they got it from sex like every other, you know, diseases, and people don’t talk about that”.

Aboriginal Health Practitioner

“Well, I actually spoke to someone with hep B and they said they thought it was sin in their blood, you know this man was quite Christian, so the thought – he thought because he had sinned, it was sin. That’s how he explained it to me, it was, sin in my blood”.

Aboriginal Health Practitioner

A participant holding cultural and social significance in the community highlighted that shame tends to be more prevalent among older than younger members of the community when it comes to the diagnosis or discussion of CHB. According to him, the older generation often places more emphasis on the responsibility of transmission of hepatitis B based on gender (females rather than males), resulting in females bearing the brunt of shame.

“The older generation, they have shame, they blames (sic) the women”.

Aboriginal Health Worker

“Specially (sic) for like, me, you know. Tradition men. The young ones, they don’t mind. We have a shame. Older generation”.

Aboriginal Community Member

Themes of shame around other blood-borne viruses, including human immunodeficiency virus (HIV), emerged. Hepatitis B and HIV share a common transmission route through bodily fluids, and the similarity in pronunciation between “hep B” and “HIV” may have led to some confusion between the two viruses.

“Yeah, it’s just similar to like HIV. You know it’s a shame job”.

Aboriginal Health Practitioner

“When you say hep B, it sounds like HIV.”

Aboriginal Community Member

Shame was also a prominent topic during conversations regarding transmitting hepatitis B from mother to child, especially regarding the perception of hepatitis B being viewed by many community members as a “woman’s disease”. This perception intensified the sense of guilt experienced by women who transmitted hepatitis B to their children. To address this issue, it was felt that providing accurate information regarding mother-to-child transmission in the app could help reduce the blame placed on women. By explaining all the other ways hepatitis B could be transmitted, the app played a crucial role in alleviating some of the shame and stigma associated with the condition.

Yes, very important because you know, like I said, our mob was thinking you get it from, you know, having sex, but they don’t know you can get it from, you know, those other things like, you know. And they’re important messages to shift that shame and stigma that is still there.

Aboriginal Health Practitioner

Additionally, there was a consensus that the app needed to be introduced to younger people, as they deemed this information essential despite their hepatitis B vaccination status and the lower prevalence of CHB in individuals born after 1990. Educating the younger generation about hepatitis B was seen to address the shame felt by the older generation.

### Perceptions of images

Participants were explicitly asked how they felt about the images in the app. The participants appreciated most of the images in the app as they helped them understand the story of hepatitis B.

“The other thing, it’s good cos (sic) the information was the story so people can see the pictures and say, oh that’s what it is, instead of just writing it. You know how you put it in the actual pictures and stuff, it helps explain it, especially when you see the blood circulating through and the pictures of where the liver actually sits in the body”.

Aboriginal Community Member

**Fig. 3:**
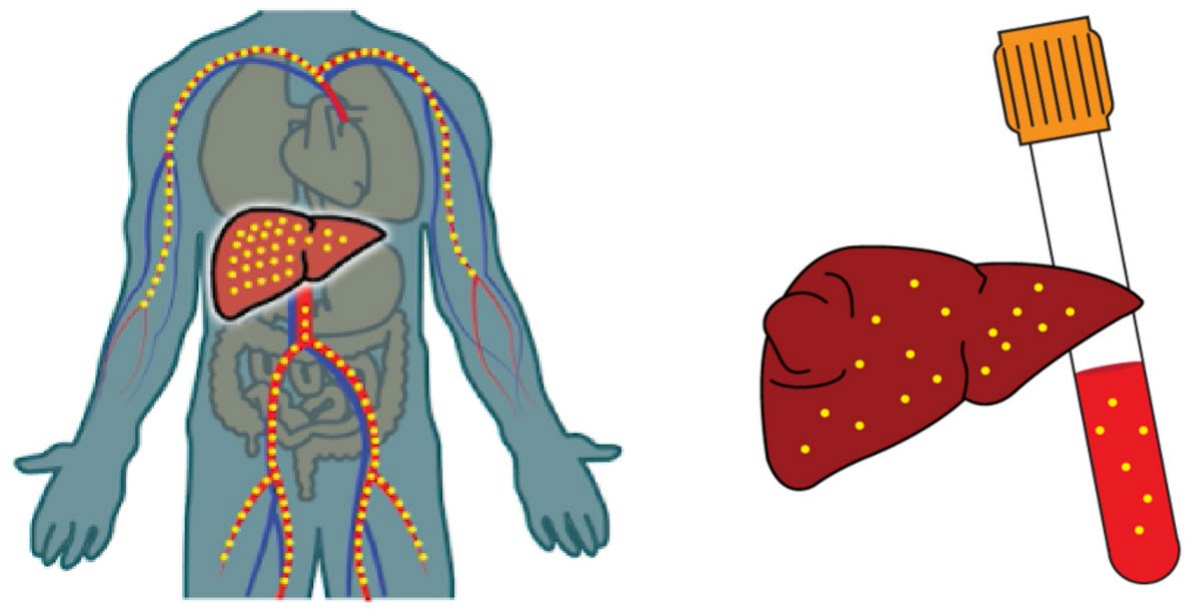
Images from the Hep B Story with yellow dots depicting hepatitis B in the liver and blood

When asked if anything in the app felt inappropriate, some participants felt the yellow dots in the pictures depicting hepatitis B (Fig. 3) could be perceived as pus and may lead to fear, resulting in a barrier from seeking care for CHB.

“Yo. It’s the ‘pus’ thing. When you - after sickness for a long time, the blood changes. The blood changes into like a pus”.

Aboriginal Health Practitioner

“They were showing that picture and they really has a pus coming out”.

Aboriginal Community Member

**Fig. 4:**
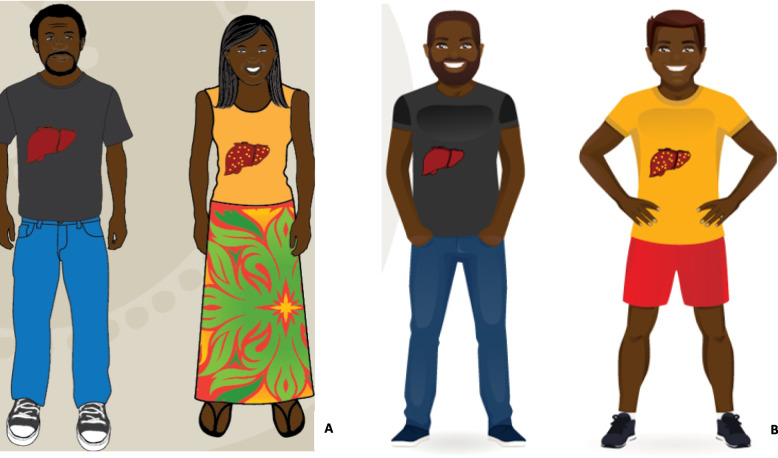
Picture of a woman with liver nodules (**A**) has been changed to a man with liver nodules (**B**)

The script was updated in the Yolŋu Matha version to negate the perception of pus in the liver and blood. Also raised in other interviews was that the females invariably had the “infected” liver in most of the images in the story. This was unintentional in the original development of the “Hep B Story” app but risked laying blame on females for transmission of hepatitis B, as hepatitis B had been viewed in some communities as a woman’s disease. The images were updated to show men with the infected liver (Fig. 4), which was considered extremely important as there was already a separate women’s business section.

**Fig. 5:**
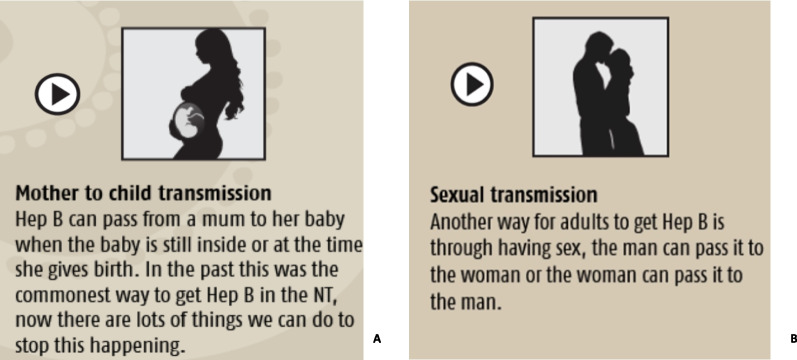
Images associated with information on **A** mother-to-child and **B** sexual transmission of hepatitis B

Two images in the “other ways to get hep B section” that created dialogue between participants were the images of the pregnant woman, Fig. 5A and the kissing couple Fig. 5B. Participants repeatedly raised that males should not view or discuss sex or women’s business, and therefore, the two figures caused unease amongst many of the male participants. This was despite the app having a separate section for women’s business.

The only part that I reckon, it’s the – I don’t know. I’m just speaking on my people, like with the pregnant part and the sexual part, that’s the only one”.

Aboriginal Community Member

“Kissing picture not good, pregnant tummy not good, rest ok”.

Aboriginal Community Member

Specifically, a discussion regarding Fig. 5B was held; given that this highlighted a potential mode of transmission between partners, it was not modified as elders felt that despite the potentially offensive image, it was essential that users of the app understood all modes of transmission of hepatitis B.

“Yo. That’s our only way of understanding or giving that education into the people, but… just to say it straight, no good picture. Part just the way of life, yes?”

Aboriginal Health Practitioner

Instead, a compromise was made to move the images of the pregnant woman and kissing couple to a separate page with a warning of the following content, allowing the app user to skip past it to ensure no offence was caused.

### Other suggestions

**Fig. 6:**
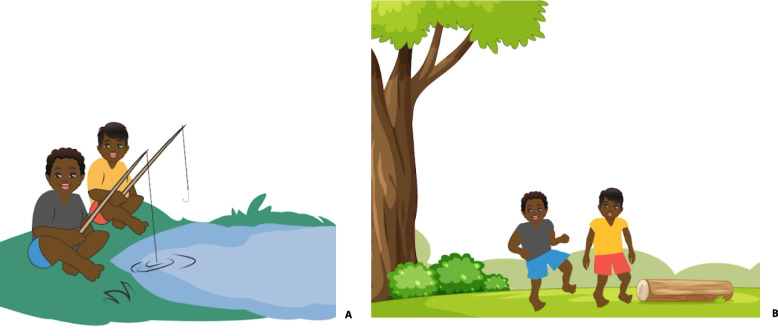
“Fishing story” (**A**) changed to two people “collecting wood” (**B**) for the Central Australian language versions

Several other adjustments were made to the “Hep B story” resulting from information from the interviews and focus groups. One example was the modification of the “Fishing story” to a story about “collecting wood” for the Central Australian languages (Fig. 6). This was because Central Australia has an arid climate, and fishing is not an activity Aboriginal people in this region undertake. Participants felt they needed to relate to the story and images to receive the message correctly.

“Need to get the story right (fishing story), different ones for the Top End and desert”.

Focus Group Participant

Several additional modifications were implemented in the app. Firstly, a warning was included at the beginning of the app, indicating that the story may contain the voices of Aboriginal individuals who have since passed away. This precaution was taken to ensure the app’s long-term usage, especially considering that most of the translation work and voice recordings were conducted by community Elders. Another modification included a message to attend the clinic and see the doctor if people were concerned about any information provided in the app. Additionally, a simple explanation was provided within the app, stating that hepatitis means inflammation of the liver, with “hepa” referring to the liver and “titis” indicating inflammation”. Notably, users were informed that hepatitis is a worldwide problem that can affect anyone.

“Explain what it is you know, saying it’s an inflamed liver because a lot of people are afraid of hep B they don’t know what it is at all”.

Aboriginal Community Member

Suggestions were put forward to enhance the volume of the app, as it was noted that while the app is audible in a clinical setting, it becomes difficult to hear in community settings with background noise such as wind, children playing, etc. Another change implemented was the introduction of the “all play” function, allowing narration to begin automatically as a new page is accessed, eliminating the need to press the play button. This adjustment aimed to assist the older generation in navigating through the story more easily.

## Discussion

This study has confirmed that the “Hep B Story” app will serve as an educational health resource for the Aboriginal population within the NT. It will also function as a tool for the health workforce to engage with culturally and linguistically diverse Aboriginal people. We have ensured it is culturally safe and acceptable for use across 11 Aboriginal language groups in the NT, encompassing approximately 70% of the NT Aboriginal population. Additionally, we have refined the app based on feedback from the focus groups, ultimately producing a better version.

Health apps have great potential in community and individual health promotion, with evidence suggesting that tailored health apps can facilitate behavioural change and aid in chronic disease management [40–42]. While approximately 250 mobile health apps are being added daily to the Google Play and Apple App stores [43] and are increasingly adopted in Aboriginal and Torres Strait Islander communities [44], evidence supporting their effectiveness is limited [45].

The evaluation of the “Hep B Story” app involved the engagement of individuals with real-world understanding and meaningful, lived connections to each of the 11 Aboriginal language groups. With a flexible interview protocol, integrating community-led story-telling or yarning initiatives, focus groups gathered the participants’ thoughts, attitudes, beliefs, and experiences. Yarning [46, 47] is a method recommended for use in Aboriginal and Torres Strait Islander health research [48, 49] and was the preferred method selected by the participants of the focus groups. The cultural safety of yarning enables the emergence of sensitive issues, fostering agency among participants, including the ability to disclose information at their own discretion [46, 50]. Participants felt at ease, and the conversation flowed freely. Each yarning session didn’t strictly adhere to an orderly script but addressed each area as the conversation wove its way around. We received precious feedback that most likely would not have been received using a traditional qualitative evaluation framework approach. Yarning, as a method of evaluating the “Hep B Story”, was also instrumental in widespread education about hepatitis B. During yarning groups, discussions extended beyond just talking about the app. Members told personal stories about how their families had been affected by hepatitis B. Participants could also discuss hepatitis B and delve into gathering more knowledge. For example, many participants were interested in how the virus integrates into the host DNA. Others wanted a detailed explanation of pathology and radiology tests. Participants could also identify the signs of liver failure in community members, with most participants knowing someone currently living with liver disease. All 95 participants felt the information about hepatitis B was so vital that they planned to pass it on to their families and encourage them to go for a checkup at the clinic. We are unable to assess the exact reach of the project’s educational component, but we anticipate it was extensive, as our focus groups were delivered NT-wide.

Kinship dynamics and relationships were crucial in shaping our approach to addressing sensitive topics within the app [12]. We recognised that discussions about health issues can be deeply intertwined with family relationships and cultural practices. Therefore, our evaluation and refinement focused on ensuring that the app content and messaging respected these dynamics. Culturally meaningful relationships between specific individuals can directly affect communication skills and ability to seek information, significantly impacting health-related behaviours and outcomes [16]. By responding to participants’ concerns and reordering the app’s layout, with warnings in appropriate places, we have negated the possibility that an individual will be exposed to information they should not be sharing culturally. Addressing the issue of blood transfer between males and females in the “fishing story” was necessary as blood has held and continues to hold diverse meanings for Aboriginal Australians, the significance of which varies across space and time [51]. Although the belief systems and practices vary by region and group, there is considerable overlap in the meanings and relationships between blood and cosmological understandings [51]. Blood is considered to be a source of power with healing properties. However, widespread sorcery beliefs and practices concerning blood also exist that, if improperly, shed a source of danger and illness [51, 52], therefore necessitating the immediate change to the fishing story.

Addressing and correcting the language and conceptual errors in the translated “Yolŋu Matha” version reduced the perception of threat, fear and offence felt by the participants. Although fear may be a motivating factor for engaging with healthcare, it can also act as a deterrent, posing a barrier to receiving care [16]. Medically accurate information must be disseminated via the app in a culturally sensitive manner. This highlights the importance of having a member of the Aboriginal Health Workforce familiar with the app’s content to clarify any misunderstandings and reinforce the critical messages. To ensure the app’s adoption, the Hep B PAST team co-designed a specific “Managing Hepatitis B for the Aboriginal Health Workforce” course [9, 10]. This course provides extensive training for the Aboriginal Health Workforce, enabling them to practice autonomously within local health clinics. Within this course, participants are trained in the use of the app.

Shame, especially in the context of relationships or images, was a recurrent point of discussion by most participants. Shame can affect multiple domains of health and subsequent health outcomes. Shame, along with culturally inappropriate services or staff at the community clinic, are known barriers to service utilisation when dealing with chronic diseases in an Indigenous context [53, 54]. Minimising stigma, shame, and blame is closely connected to providing education in one’s first language and engaging Aboriginal health and research personnel in the conversations [12, 55]. Great emphasis needs to be placed on addressing shame around hepatitis B.

The outcome was successful, knowing the app would be largely culturally appropriate and medically accurate. A consultation process that instils community knowledge and develops relationships based on trust and respect is crucial for developing and evaluating such apps. The app’s contents can be further improved by having regular interactions with the patients and healthcare workforce from the community to ensure no technical or culturally inappropriate issues have been identified. Tailoring health promotion programs to meet the needs of Aboriginal and Torres Strait Islander communities is essential, considering their diverse languages and unique perspectives on health and wellbeing [56]. For Aboriginal and Torres Strait Islander peoples, health is viewed holistically, encompassing physical, emotional, social, and spiritual aspects. Healthcare services must aim for individuals to achieve their full potential and promote the overall well-being of their communities [57]. This highlights the importance of co-developing technology with, as opposed to for, Aboriginal and Torres Strait Islander communities. Involving users, caregivers, health professionals, and Elders in the design process ensures that the technology is relevant and valuable. A robust engagement model is crucial for successful technology adoption, emphasising meaningful community involvement throughout all developmental and testing phases [44, 58].

## Conclusion

A rigorous process was used to develop the initial version of the “Hep B Story” app; however, a “real-life” evaluation and testing was necessary before creating the additional ten language versions. Using a PAR approach blended with Indigenous research methods, this evaluation allowed us to identify errors, thereby resulting in the development of an updated and enhanced version of the app. An iterative approach and strong community engagement were essential to ensure the app was culturally safe and appropriate. Allowing sufficient time for the development and evaluation of apps is crucial. We also recommend avoiding the use of knowledge-based evaluations in an Aboriginal setting to ensure relevant and culturally appropriate feedback is obtained.

### Supplementary Information

Supplementary Material 1.

Supplementary Material 2.

## Data Availability

The data generated and analysed during the current study are not publicly available due to ethical and privacy considerations but may be available from the corresponding author at a reasonable request, pending approval granted by the Hep B PAST steering committee and Menzies Infectious Diseases IRG.

## Abbreviations

CHB: Chronic Hepatitis B
HBV: Hepatitis B Virus
IRG: Indigenous Reference Group
NT: Northern Territory
PAR: Participatory Action Research
WHO: World Health Organization
