# Supplementary material for: An evaluation and refinement of the “Hep B Story” app, tailored to meet the community’s cultural needs
Source: BMC Health Serv Res. 2024 Jun 7;24:710. doi: 10.1186/s12913-024-11149-y (PMC11162029; doi:10.1186/s12913-024-11149-y)
Supplement: Supplementary file 1 — Supplementary Material 1. [file 12913_2024_11149_MOESM1_ESM.pdf]

Hep B PAST Partnership Members and Individuals

Partner Organisations

Menzies School of Health Research
Northern Territory Government, NT Health
ASHM
Katherine West Health Board Aboriginal Corporation
Miwatj Health Aboriginal Corporation
NT AIDS and Hepatitis Council, NTAHC
Mala'la Health Service Aboriginal Corporation
Marthakal Homelands Health Service
Central Australian Aboriginal Congress

Associate Partner Organisations

WHO Collaborating Centre for Viral Hepatitis, VIDRL, Doherty Institute
Victorian Infectious Diseases Reference Laboratory - VIDRL
Hepatitis Australia

Investigators and Contributors

Jane Davies	Menzies School of Health Research and NT Health	CI
Sarah Mariyalawuy Bukulatjpi	Miwatj Health Aboriginal Corporation	CI
Christine Connors	NT Health	CI
Joshua Davis	Menzies School of Health Research	CI
Robert Batey	NT Health	CI
Benjamin Cowie	WHO Collaborating Centre for Viral Hepatitis, VIDRL, Doherty Institute	CI
Steven Tong	Doherty Institute for Infection and Immunity	CI
Anna Ralph	Menzies School of Health Research	CI
Adrian Miller	Central Queensland University	CI
Kelly Hosking	NT Health and Menzies School of Health Research	AI
George Garambaka Gurruwiwi	Menzies School of Health Research	AI
Roslyn Dhurrkay	Miwatj Health Aboriginal Corporation	AI
Belinda Greenwood-Smith	NT Health	AI
Catherine Marshall	NT Health	AI
Geoffrey Stewart	NT Health	AI
Nicole Allard	WHO Collaborating Centre for Viral Hepatitis, VIDRL, Doherty Institute	AI
Manoji Gunthilake	NT Health	AI
Vicki Krause	NT Health	AI
Ashleigh Qama	WHO Collaborating Centre for Viral Hepatitis, VIDRL, Doherty Institute	PI
Karen Fuller	Katherine West Health Board Aboriginal Corporation	PI
Jessica Michaels	ASHM	PI

Lou Sanderson	Miwatj Health Aboriginal Corporation	PI
Phillip Merrdi Wilson	NT Health	PI
Genevieve Dally	NTAHC	PI
Kerrie Jordan	NTAHC	PI
John Boffa	Central Australian Aboriginal Congress	PI
Alexis Apostolellis	ASHM	P
Amanda Dhagapan	Miwatj Health Aboriginal Corporation	P
Anna Deng	WHO Collaborating Centre for Viral Hepatitis, VIDRL, Doherty Institute	P
Anngie Everitt	Menzies School of Health Research	P
Barbara De Graaff	Menzies Institute for Medical Research	P
Brianna Summers	Katherine West Health Board Aboriginal Corporation	P
Carrie Fowler	Hepatitis Australia	P
Catherine Blacker	NT Health and Menzies School of Health Research	P
Catherine Gargan	NT Health	P
Catherine Stoddart	NT Health	P
Charles Pain	NT Health	P
Cheryl Ross	Menzies School of Health Research	P
David McGuinness	Katherine West Health Board Aboriginal Corporation	P
David Reeve	NT Health	P
Diane Hampton	Katherine West Health Board Aboriginal Corporation	P
Eddie Mulholland	Miwatj Health Aboriginal Corporation	P
Ella Meumann	NT Health	P
Elizabeth Coombes	NT Health	
Emily Vintour-Cesar	Menzies School of Health Research	P
Emma Childs	NT Health and Miwatj Health Aboriginal Corporation	P
Hayden Jose	ASHM	P
Hilary Bloomfield	Miwatj Health Aboriginal Corporation	P
Hugh Heggie	NT Health	P
Isabelle Purcell	ASHM	P
Jaclyn Tate-Baker	NT Health	P
Jayne Porter	NT Health	P
Jyoti Jadeja	NTAHC	P
Katherine McNamara	NT Health	P
Katie McGuire	Menzies School of Health Research	P
Keith Forrest	Mala'la Health Service Aboriginal Corporation	P
Kelly Banz	NT Health	P
Kelly-Anne Stuart-Carter	NT Health	P
Khim Tan	NT Health	P
Leanne O'Connor	NT Health	P
Lesley Scott	NT Health	P
Letisha Murray	NT Health	P
Levinia Crooks^	ASHM	P
Linda Bunn	NT Health	P
Lorraine Johns	Katherine West Health Board Aboriginal Corporation	P
Lucie Perrisel	ASHM	P
Marco Briceno	NT Health	P
Margaret Littlejohn	Doherty Institute for Infection and Immunity	P
Maria Scarlett	NTAHC	P

Marilou Capati	Top End Medical Centre	P
Matthew Maddison	NT Health	P
Melita McKinnon	Menzies School of Health Research	P
Mikaela Mobsby	NT Health	P
Molly Shorthouse	Miwatj Health Aboriginal Corporation	P
Natasha Tatipata	NT Health	P
Nicole Romero	WHO Collaborating Centre for Viral Hepatitis, VIDRL, Doherty Institute	P
Paula Binks	Menzies School of Health Research	P
Penny Ramsey	NT Health	P
Peter Markey	NT Health	P
Peter Nihill	NT Health	P
Phoebe Schroder	ASHM	P
Prashanti Manchikanti	Miwatj Health Aboriginal Corporation	P
Rebecca Katiforis	NT Health	P
Robyn Liddle	Menzies School of Health Research	P
Rosalind Webby	NT Health	P
Richard Sullivan	Menzies School of Health Research	P
Sami Stewart	ASHM	P
Sandra Nelson	NT Health	P
Sean Heffernan	Katherine West Health Board Aboriginal Corporation	P
Sean Taylor	NT Health	P
Shiraline Wurrawilya	NT Health	P
Sinon Cooney	Katherine West Health Board Aboriginal Corporation	P
Sonja Hill	ASHM	P
Stephen Locarnini	Doherty Institute for Infection and Immunity	P
Steven Skov	NT Health	P
Su Govindasamy	ASHM	P
Sudharsan Venkatesan	Menzies School of Health Research and NT Health	P
Tammy-Allyn Fernandes	NT Health	P
Tanya Plavins	Central Australian Aboriginal Congress	P
Teresa De Santis	NT Health	P
Terese Ngurruwuthun	Miwatj Health Aboriginal Corporation	P
Tiana Alley	Menzies School of Health Research	P
Timothy Nabegeyo	NT Health	P
Vanessa Towell	ASHM	P
Wendy Page	Miwatj Health Aboriginal Corporation	P

Key: CI - Chief Investigator, AI- Associate Investigator, PI – Partner Investigator, P – Partner, ^ - Deceased

Contact person: Jane Davies - Jane.davies@menzies.edu.au
