# Supplementary material for: An evaluation and refinement of the “Hep B Story” app, tailored to meet the community’s cultural needs
Source: BMC Health Serv Res. 2024 Jun 7;24:710. doi: 10.1186/s12913-024-11149-y (PMC11162029; doi:10.1186/s12913-024-11149-y)
Supplement: Supplementary file 2 — Supplementary Material 2. [file 12913_2024_11149_MOESM2_ESM.pdf]

Semi-structured interview and community focus group guide – "Hep B Story" app evaluation.

**Reiterate confidentiality; it is not a test, just exploring ideas, with no consequences. Feel free to speak openly and honestly and ask as many questions as they would like to.**	
• Can you tell me a bit about yourself, where you live, what you do<br>• Gender/age/remoteness<br>• Role within family, community, work<br>• Number of languages spoken<br>• First language	Background of each individual.
• What do you understand by the phrase Hepatitis B infection? **(If no knowledge, move directly to the next section)**<br>• What do you think it is/does?<br>• Where did this knowledge come from?<br>• Your perception of the general Community's knowledge?<br>• Does it concern you?<br>• What harm, if any, do you think it causes?<br>• How do you think you get Hepatitis B?<br>• Is it a problem for you or your family?	Hepatitis B knowledge.
• What do you think of the Hep B Story app?<br>• Is it helpful?<br>• Do you think the app contains the right amount of information?<br>• Too much?<br>• Too little?<br>• Anything missing?<br>• Anything that shouldn't be there?<br>• Anything about the images that are not appropriate for your language?<br>• Anything about the content that is not appropriate for your language?	Thoughts about the app content.
• Do you think the app is culturally appropriate for most Aboriginal people in the NT?<br>• Is there anything in the app that might offend Aboriginal people who speak your language?<br>• Would you like to see the app in your language?	Cultural Safety.
• Do you think people want to understand more about hepatitis B?<br>• Do you think it is helpful for people to understand more?	Attitudes towards Western health knowledge.
• Do you think it would help people to have the app in your language?<br>• Do you think it will improve people's knowledge?<br>• Do you think it will make people more likely to go to the clinic to get care for their hepatitis B?<br>• Are you aware of any shame or stigma about hepatitis B – if so, do you think the app could help change this?	Translation into other languages.
